# Re-Enactment as a Method to Reproduce Real-World Fall Events Using Inertial Sensor Data: Development and Usability Study

**DOI:** 10.2196/13961

**Published:** 2020-04-03

**Authors:** Kim Sarah Sczuka, Lars Schwickert, Clemens Becker, Jochen Klenk

**Affiliations:** 1 Department of Clinical Gerontology Robert-Bosch-Hospital Stuttgart Germany; 2 Institute of Epidemiology and Medical Biometry Ulm University Ulm Germany; 3 IB University for Applied Science Berlin Study Center Stuttgart Stuttgart Germany

**Keywords:** falls, simulation, inertial sensor, method

## Abstract

**Background:**

Falls are a common health problem, which in the worst cases can lead to death. To develop reliable fall detection algorithms as well as suitable prevention interventions, it is important to understand circumstances and characteristics of real-world fall events. Although falls are common, they are seldom observed, and reports are often biased. Wearable inertial sensors provide an objective approach to capture real-world fall signals. However, it is difficult to directly derive visualization and interpretation of body movements from the fall signals, and corresponding video data is rarely available.

**Objective:**

The re-enactment method uses available information from inertial sensors to simulate fall events, replicate the data, validate the simulation, and thereby enable a more precise description of the fall event. The aim of this paper is to describe this method and demonstrate the validity of the re-enactment approach.

**Methods:**

Real-world fall data, measured by inertial sensors attached to the lower back, were selected from the Fall Repository for the Design of Smart and Self-Adaptive Environments Prolonging Independent Living (FARSEEING) database. We focused on well-described fall events such as stumbling to be re-enacted under safe conditions in a laboratory setting. For the purposes of exemplification, we selected the acceleration signal of one fall event to establish a detailed simulation protocol based on identified postures and trunk movement sequences. The subsequent re-enactment experiments were recorded with comparable inertial sensor configurations as well as synchronized video cameras to analyze the movement behavior in detail. The re-enacted sensor signals were then compared with the real-world signals to adapt the protocol and repeat the re-enactment method if necessary. The similarity between the simulated and the real-world fall signals was analyzed with a dynamic time warping algorithm, which enables the comparison of two temporal sequences varying in speed and timing.

**Results:**

A fall example from the FARSEEING database was used to show the feasibility of producing a similar sensor signal with the re-enactment method. Although fall events were heterogeneous concerning chronological sequence and curve progression, it was possible to reproduce a good approximation of the motion of a person’s center of mass during fall events based on the available sensor information.

**Conclusions:**

Re-enactment is a promising method to understand and visualize the biomechanics of inertial sensor-recorded real-world falls when performed in a suitable setup, especially if video data is not available.

## Introduction

Falls are a common health problem that can lead to serious physical consequences such as fractures, reduced quality of life, loss of independence, and institutionalization. Furthermore, fall-related injuries seriously increase mortality in older persons [1]. One-third of community-dwelling people older than 65 years fall at least once a year, and half of them fall more than once [2]. Besides the individuals’ health burden, falls also have a major social and economic effect with annual costs accounting for 0.85% to 1.5% of the total health care expenditures [3].

Meta-analyses identified about 30 fall risk factors in community-dwelling older persons [4]. These risks include fall history as well as balance and gait problems. Nevertheless, fall risk prediction models show limited performance, which suggests that we do not fully understand the complex interplay of factors triggering fall events. This may be because information about fall events are mainly derived from subjective reports by fallers or proxies, which can be biased in many ways [5]. Lack of reporting or false reporting can be related to cognitive impairment of the subjects, shame of reporting and fear of consequences, or simply due to difficulties in defining a fall [6]. Objective information is rare and, therefore, many aspects including fall-related activities, environmental factors and movement patterns before, during, and after the falling phase remain unclear. Body-worn sensor technology might enhance our understanding of falls and thereby also lead to more effective methods for fall prevention, fall risk assessment, and fall detection. With the rapid development of eHealth, small wearable devices such as body-worn sensor technology can provide objective measures of physical activity and the kinematics of human movement [7].

Although falls are common, it is challenging to capture real-world fall signals due to the long observation period and the limited recording duration of sensor devices. The Fall Repository for the Design of Smart and Self-Adaptive Environments Prolonging Independent Living (FARSEEING) consortium, funded by the seventh European Union Framework Program for Research, has been able to capture and validate real-world falls of older people who have an increased risk of falling, measured by body-worn sensor technology [8]. Analysis of these fall signals showed, for example, that the characteristics presented by inertial sensor measurements are relevant to improve the understanding of the postimpact phase [9]. Movement patterns during the ground phase were different between fall events with and without successful recovery to a standing position. These findings are important for redesigning emergency response processes after falls to better support individuals in cases of an unrecovered fall.

Even though the signals provide precise measures for acceleration, angular velocity, and magnetic north, it is not possible to directly derive visualization and interpretation of movements during a fall event. In contrast, video data facilitates the possibility of estimating the kinematics of falls as well as the fall-related movements before and after the fall event [10]. However, analyzing the complex movement patterns of fallers from planar video data is challenging, due to motions of body segments that are out of the plane or occluded [11]. Furthermore, due to privacy issues, it is usually not possible to capture video data during everyday life. In the absence of well-described real-world fall recordings and due to the huge effort in recording objective fall data, researchers have tried to bridge the knowledge gaps by simulating fall events. However, comparisons between acceleration signals of simulated and real-world fall events has shown that there are considerable differences [12]. This might be due to a lack of real-world data for designing a more suitable and realistic simulation protocol. For example, the preimpact phase was excluded from simulations, but analysis of this phase could identify protective movements or provide a better understanding of the circumstances that lead to a fall. Furthermore, if the simulated fall was self-initiated, the movement pattern differed a lot from the movement pattern of the real-world fall, because the volunteers did not know how to fall in a realistic way. It was shown that the acceleration values were closer to those of real-world fall events when the subjects were forced to fall by releasing them suddenly from a backward lean with the instruction to avoid a fall [12]. To perform more suitable simulations, it is essential to find methods to create a realistic experimental protocol that can be easily reproduced. The obtained information could be of great value and give insight into the causes of falls as well as what happened during the fall. To the best of our knowledge there is no current method to obtain this kind of information based on sensor signals without having another information source such as video. However, such information could help better predict falls and develop new fall prevention interventions as well as fall detection approaches.

Connell and Wolf [13] previously proposed a re-enactment method to validate subjective fall reports. Participants were interviewed and asked to re-enact in detail (if they felt comfortable) all activities, body movements, body part placements, and interactions with the environment at the location of the incident to obtain more precise information. This method of re-enactment is a promising approach to improve simulation protocols and to produce more realistic fall simulations. We adapted the re-enactment method to visualize and enhance the interpretation of sensor signals. The aim of this study was to describe this adapted method and to demonstrate the validity of the re-enactment approach by means of a selected common fall example.

## Methods

### Real-World Fall Data

Real-world fall data measured by inertial sensors during everyday life were obtained from subjects in different settings (eg, community-dwelling, geriatric rehabilitation) and different populations with moderate to high risk of falling (eg, Parkinson disease, cerebellar and sensory ataxia). All fall events were stored in the FARSEEING database [8]. The process of data collection by combining different sources was approved by the Ethics Committee of the University of Tübingen (495/2012BO2) and the data protection office of the Federal State of Baden-Württemberg, Germany (T 1500/231). The large number of real-world fall events within the FARSEEING database facilitated the comparisons of sensor signals that represented similar curve progressions as well as the confirmation of the feasibility to apply the re-enactment method for several diverse fall paradigms. For the purposes of exemplification, we selected one fall event of a female patient (42 years of age, height=154 cm, weight=60 kg, Montreal Cognitive Assessment=27 [14], Timed Up-and-Go=17.72 seconds [15], Short Physical Performance Battery=9 [16]) with ataxia that presented a reliable fall report and sensor signal. The corresponding fall report described the event as a forward fall initiated by stumbling over the entrance door sill. Analysis and interpretation of the triaxial sensor signal concerning movement patterns and curve progression confirmed the fall description. Based on the collected signals from the repository, the selected fall signal represents a common fall paradigm that corresponds with everyday life situations.

### Data Processing

Data acquisition was performed during the patient monitoring as well as during the re-enactment experiments using the Samsung Galaxy (SG) S3 smartphone worn on the lower back at the lumbar position (L5) with a belt, close to the center of mass. The smartphone includes a triaxial accelerometer (2 g SGS3) sampled at 100 Hz. Data were stored for off-line analysis on the smartphone. Orientation was defined as follows: z=vertical, y=mediolateral, and x=sagittal. Additionally, the re-enactment experiments were captured with a video camera (SGS8, 200-Hz sampling rate) to analyze the movements in detail. For this purpose, the video data and the sensor signal were synchronized to assign specific postures, as seen from the video tape, to each frame of the acceleration signal.

### Re-Enactment Protocol

**Figure 1:**
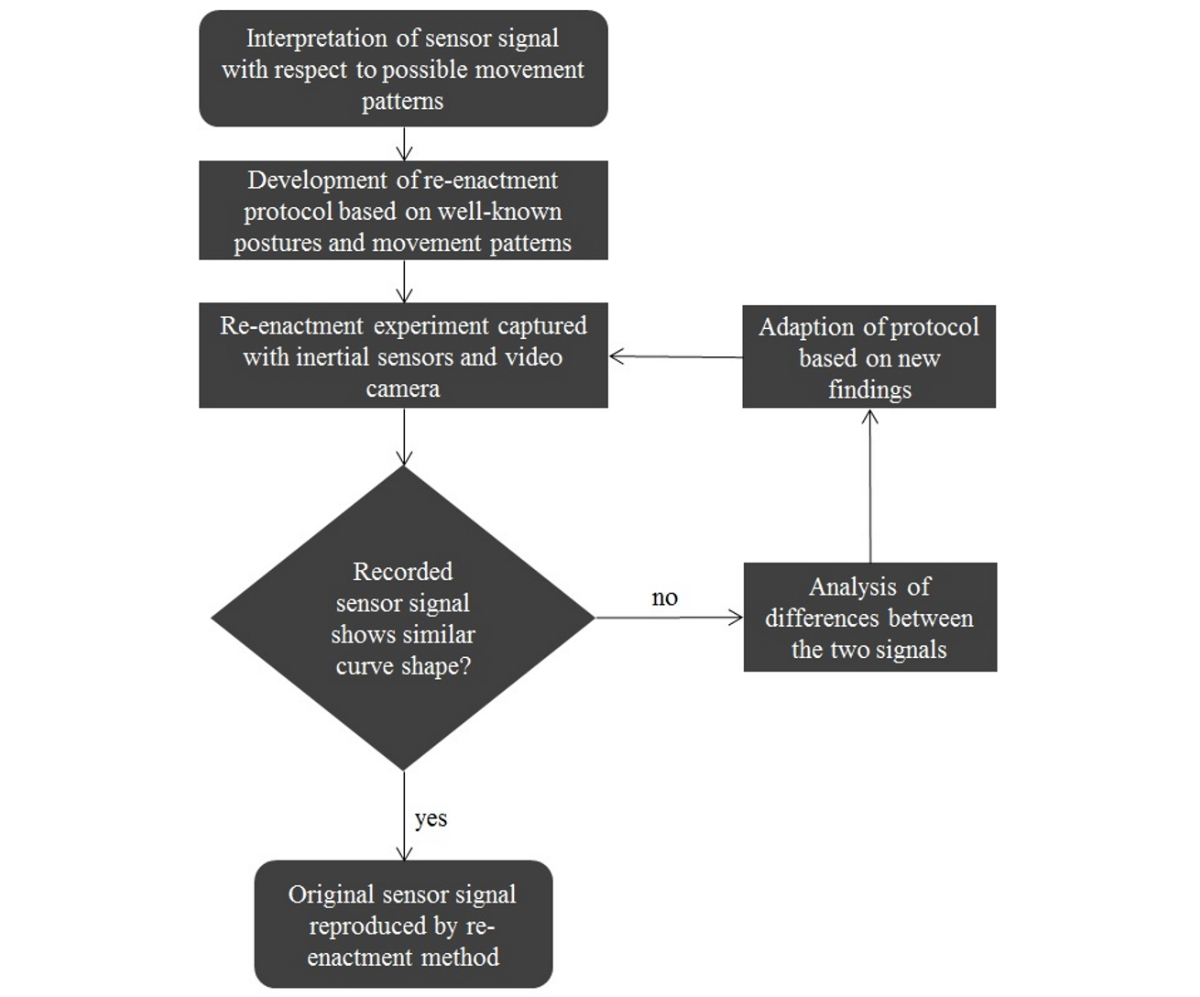
Flowchart of the re-enactment method.

Aiming to establish a simulation protocol, the selected fall signal was analyzed with regard to the movement patterns during the prefall phase (5-10 seconds before impact), falling phase, and impact phase as well as the resting and recovery phase with the faller achieving an upright standing position [17]. Based on the signal interpretation, a simulation protocol including identified postures and movement sequences was established. The protocol was conducted by an expert (woman, 28 years of age, height=168 cm, weight=61 kg, healthy, and physically active) in analyzing sensor signals of real-world fall events. Simulations started with the prefall activity and ended with the person standing upright subsequent to the impact phase. The re-enactment method was conducted under safe conditions in a laboratory setting using protective layers of mattresses to reduce the impact and avoid injuries. With the aim of producing a simulated acceleration signal similar to the real-world fall signal the protocol was performed several times. Subsequently, the re-enacted signals were compared to the real-world fall signal, and the protocol was adapted based on the findings after the first re-enactment experiment. Special attention was paid to the z component, as it indicated the motion in vertical direction as well as bending movements of the trunk section. The adapted protocol was conducted again and the newly recorded signals were compared to the original signal. In cases of new findings, the protocol was adapted again as a basis for a further trial. This re-enactment process is visualized in Figure 1. The adaptation of the protocol was repeated until the experiment led to satisfying results that showed a similar curve shape compared to the original signal from the real-world fall event.

### Validation of Re-Enactment Method

The resemblance between the simulated and the real-world fall signals was analyzed using a dynamic time warping (DTW) algorithm, which enables the comparison of two temporal sequences varying in speed and timing. The DTW algorithm compensated the temporal differences between the sensor signals. Both time series were aligned by stretching the two vectors to minimize the sum of the Euclidean distances between the corresponding points. DTW alignment was processed in R-3.4.2 (R Foundation for Statistical Computing, Vienna, Austria) with the slope-constrained step pattern “asymmetricP1” published by Sakoe and Chiba [18] with open start and open end to achieve time normalization by transforming the time axis of the real-world fall signal pattern (query) onto that of the re-enacted one (template). The slope constraining factor P=1 was chosen due to its best recognition performance, which was also shown in the study of Sakoe and Chiba [18]. Similarity between the curves was calculated by the normalized distance as defined by the Euclidean distance divided by the number of samples in the query.

## Results

**Figure 2:**
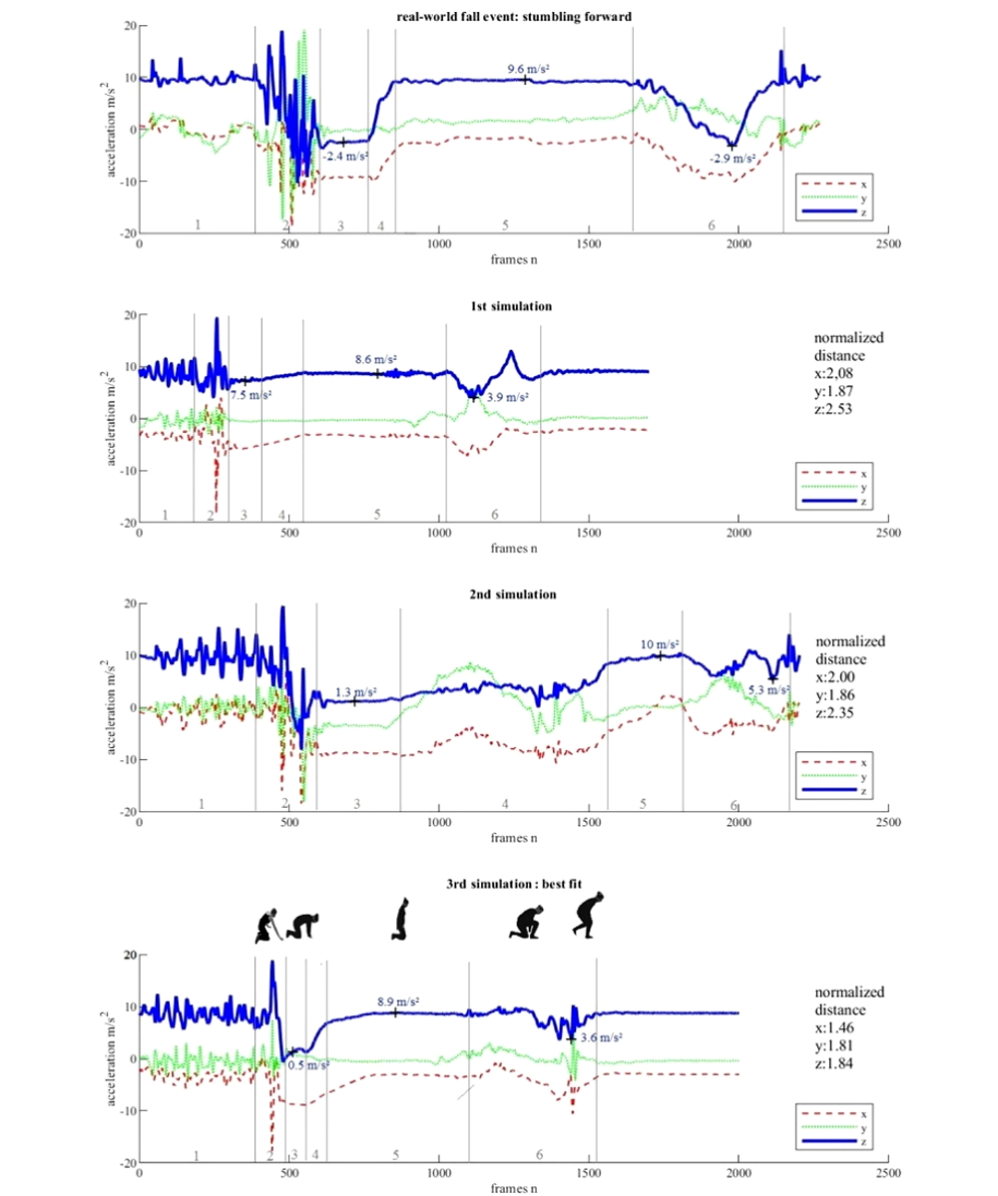
Triaxial acceleration signals of a real-world fall event and signals stepwise derived during the re-enactment experiments. Numbers 1 to 6 indicate the particular phases of the fall event (1: prefall phase with steps, 2: stumbling, falling, and impact, 3: resting, 4: raising upper body, 5: resting, 6: straighten up into standing position).

Figure 2 shows the triaxial acceleration signal of the real-world fall event at the top, and the three acceleration signals recorded during the re-enactment experiments below. The movement sequence of the chosen real-world fall event was as follows: standing unsteady, stumbling over doorsill, falling forward on the knees, and standing up again. The first simulation followed these rough instructions and the result was not satisfying, demonstrated by the second plot from the top. The impact in section 2 could not be simulated realistically due to the soft mattress, and the frequency of the steps in section 1. Focusing on the z component (vertical), the original fall signal showed a steeper slope after the impact compared to the signal of the first simulation. The acceleration values of the simulated signal in section 3 were higher as well as the value of the local minimum in section 6; whereas the values in section 5 were lower compared to the signal of the real-world fall event. Furthermore, the lifting of the upper body in section 4 was barely visible. However, the curve shape in section 3, 5, and 6 was analogous to those of the real-world fall signal. The normalized distance was 2.53, which was the highest value in all experiments. The x component of the first simulated sensor signal showed a similar curve progression in sections 3, 5, and 6 but differed regarding the acceleration values in comparison to the real-world fall signal. For the y component, the curve shape was quite similar compared with the real-world fall signal with slight deviations in section 6. Subsequently, the protocol was adapted: more pronounced steps (section 1), bending forward while kneeling (section 3), and resting in this posture for a short time period.

The second simulation produced more similar acceleration values but was still too high for the z component in sections 3 and 6. The forward bending of the upper body while standing up was not pronounced enough, which was illustrated by the minimal value of the z component in section 6. Furthermore, the z component in section 6 showed two local minima instead of one as shown in the signal of the real-world fall. Section 5 especially showed a time period that was too short. Noticeable was section 4, which was about 7 times longer and included a lot more motion in all three axes compared to the real-world fall event. The x component improved within section 3 and 6, but section 4 and 5 remained different in acceleration values and curve progression. For the y component the curve shape had to be improved, especially in section 4. However, the normalized Euclidean distance was lower for all three axes, indicating a general improvement of the simulation.

The findings were added to the protocol, and the experiment was repeated with the following instructions: scuttling or less pronounced steps (section 1), stumbling and falling forward on the knees (section 2), bending the upper body forward and touching the ground with the hands for 1 to 2 seconds (section 3), sudden raising (section 4), 4 seconds of resting on the knees with upper body upright (section 5), placing the left foot on the floor and standing up without the upper body swaying (section 6).

The resulting signal of the third simulation experiment is the lowermost subplot in Figure 2. With exception of section 1 and 2, all three axes showed very similar curve shapes compared with the real-world fall signal. The visual similarity was confirmed by the decreased normalized Euclidean distance for all three axes. The lowest distance was at 1.46, calculated for the x component. The x and z component showed a nearly 30% reduction of the normalized Euclidean distance; although the bending in section 6 could have been more pronounced for the z and x component, and the whole simulated signal was about 6 seconds shorter.

**Figure 3:**
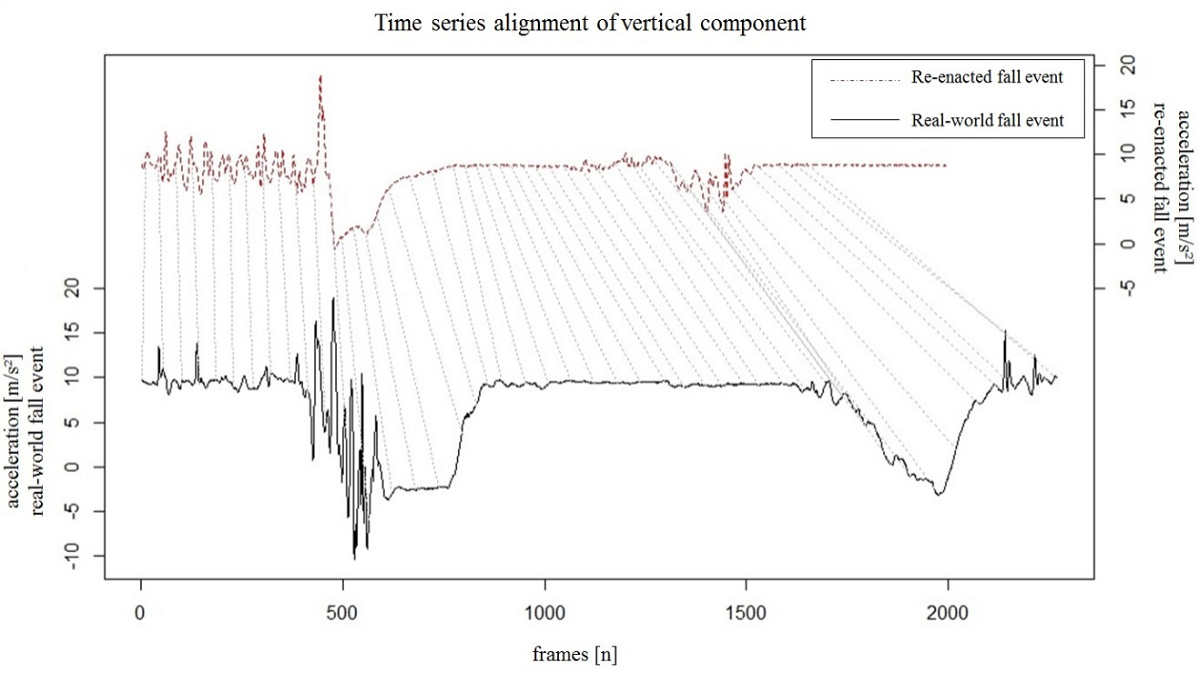
Time series alignment of the vertical acceleration signals for the real-world fall event as well as the best-fit re-enacted signal.

Figure 3 illustrates the alignment of the vertical acceleration (z) component of the real-world fall acceleration signal and the signal obtained during the third simulation. The appropriate movement sequences were linked, but there was a temporal delay resulting from the shorter duration of the re-enacted fall signal.

**Figure 4:**
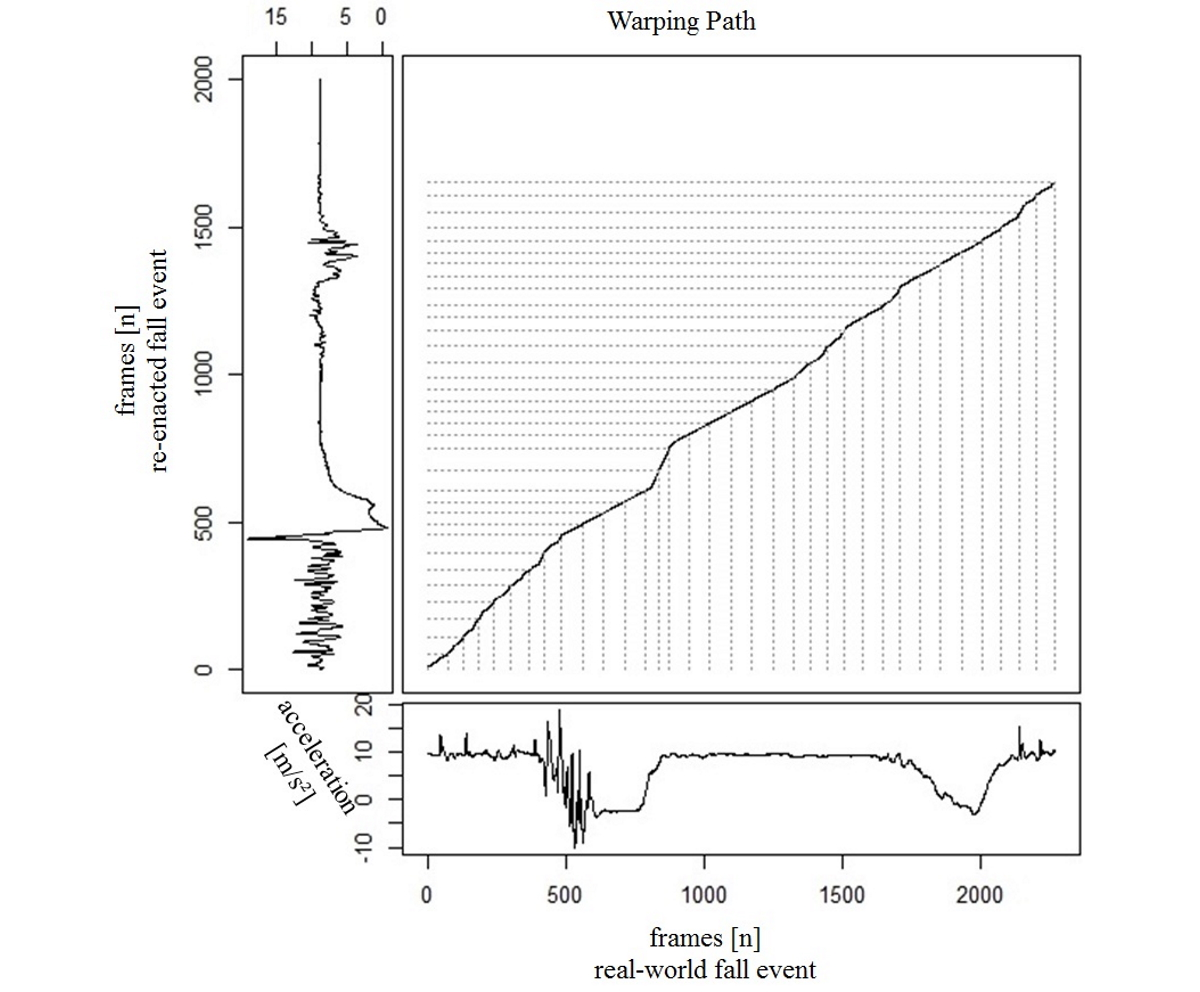
Time series alignment and warping path calculated using a dynamic time warping algorithm for the vertical component of the real-world and re-enacted fall signal.

Figure 4 displays the warping path for the vertical (z) component of the real-world fall signal and the re-enacted fall signal derived from accelerometer data. Only one part of the real-world fall signal located around frame 800 showed a dominant discrepancy and led to a sharp bend in the line because of the shorter resting phase (section 5, Figure 2) in the re-enacted signal. The DTW algorithm compensated the resulting differences in length within this section by stretching this part of the signal in the re-enacted fall event by repeating each element as many times as necessary.

## Discussion

### Principle Findings

Re-enactment was demonstrated as a suitable approach to provide new insight into real-world fall events recorded with inertial sensors, as well as fall events in general. It was possible to simulate fall events more realistically and thereby verify the interpretation even though falls were heterogeneous and showed a high variability due to the 3D movements of the fallers.

To the best of our knowledge, the introduced method is a new approach to reproduce with good approximation the inertial sensor signals of real-world fall events, which allows for more valid fall simulations under safe laboratory conditions. Prior re-enactment studies already showed that this method is adequate to conduct an examination of behavioral and environmental circumstances associated with falls [13]. We modified this approach by enhancing the written fall report with the corresponding real-world fall signal. Reproducing the sensor signal by re-enactment and additional videotaping of the simulation seems to be a suitable method to prove the interpretation of a real-world fall event when video data is missing. By synchronizing video and sensor data, every change in curve progression of the sensor signal can be associated with a specific posture or movement pattern and the circumstances can be retraced more easily.

Comparison between real-world and re-enacted fall signals was performed by applying a DTW algorithm. This method was suitable to measure the similarity between two events of different lengths in the time series. This was an important finding since our experiments demonstrated that it was impossible to simulate fall events chronologically with enough precision in the exact timing of each specific posture. Most other correlation approaches cannot handle this problem. It was further shown that the applied DTW algorithm was able to stretch the signals in a way that enables the characteristic patterns to be assigned to each other. There is no natural threshold for the normalized distance to show an appropriate fit. However, previous studies already indicated that DTW was a suitable approach with regard to pattern recognition in human motion regardless of thresholds [19-22]. Our results also suggest that the DTW approach might be a promising method to detect entire fall patterns or at least parts of the events from accelerometer data. This could also help to further improve fall detection algorithms.

Our results show that the re-enactment method provides a tool to understand fall-related movements. Based on the FARSEEING database, it will be possible to build up a database with sequences of sensor signals that represent specific movement patterns and link the corresponding pictures or video data that show the re-enacted movement. Using such a database in combination with the method of DTW could help to identify movement sequences of newly acquired fall signals and thereby improve the understanding of falls, including the causes and consequences. With this new knowledge it will be possible to develop more reliable fall risk models [23].

The FARSEEING database contains about 200 well-described fall events [8]; however, realistic simulations are still necessary. It seems unrealistic to collect a sufficient number of real-world falls for all open research questions and data intensive analytic methods such as automated machine learning approaches for fall detection. Currently, fall detection is insufficient, such as when implemented in home alarm systems. The main reason seems to be unrealistic simulated falls by younger subjects with insufficient knowledge of real-world falls [12]. Applying such fall detection algorithms to real-world situations resulted in high rates of false positive or false negative alarms [24]. The application of data on real-world falls from the FARSEEING database in the algorithm development highly improved the detection performance [25]. However, there is still an unacceptable high false alarm rate. Machine-learning approaches already show promising results in activity recognition based on data from waist-worn inertial sensors [26,27] and might further improve the results, but would need additional realistic fall data.

The re-enactment method will facilitate and enhance the quality of fall simulation to provide more realistic data input for algorithm developers. Volunteers could be trained to simulate real-world falls that were sufficiently similar using re-enacted fall signal data. As a next step, a study is planned to systematically re-enact the falls provided by the FARSEEING database and to analyze the repeatability of the results when re-enactment is performed by different persons.

### Strengths and Limitations

This new approach was developed and validated to broaden the knowledge of real-world fall events and close the information gap in cases of missing or fragmentary fall description. A remarkable strength of this study is the development of the re-enactment method based on real-world fall data derived from the FARSEEING meta database, which is currently the largest collection of real-world fall events recorded with inertial sensors [8]. Although fall events are heterogeneous, it was possible to compare several real-world data sets with a similar fall scenario and to identify fall paradigm-specific patterns, which could be replicated within the re-enactment protocol and lead to realistic simulations. Furthermore, it seems that re-enactment can be performed by any person able to simulate the fall event without matching the clinical characteristics of the original faller. Shawen et al [28] even demonstrated that a fall detection approach based on the inertial sensor signals of healthy participants recorded with a smartphone can be used to resemble characteristics of other populations, such as individuals using prostheses. Even though we introduced the re-enactment method by means of only one exemplary real-world fall event, this new approach is feasible for several real-world fall data with similar results as shown. Further examples can be found in the Multimedia Appendicies 1-3.

However, this method has several limitations. Due to safety conditions the re-enactment experiments had to be performed with constraints on the impact phase. A soft mattress was used to lessen the impact during re-enactment experiments. Therefore, the acceleration values caused by the impact differ from those of real-world fall signals. Nevertheless, the reliable alignment of both signals can be seen in Figure 3. The DTW algorithm that was used is able to match the signals on the basis of a similar curve progression, despite the values in the original and re-enacted signals differing in the relatively short part of the impact phase. However, when focusing on the impact phase it will be necessary to use signals derived from real-world falls with sufficient ranges of acceleration. It was previously shown that signals derived from simulated impacts were not acceptable, at least when fall detection algorithms were evaluated [29].

Even though the simulation of fall events, including all phases, is feasible in general, less dominant motions might have been overlooked and not reproduced by re-enactment. Due to the fact that no video data were available from the real-world fall events, the re-enacted movements were impossible to verify. In addition, the fall reports were often limited and provided little to no information concerning movement behavior and circumstances.

Furthermore, we only performed the re-enactment method for common fall paradigms such as stumbling over a doorsill or falling backwards while opening a door. It seems that these paradigms can be reliably simulated in a laboratory. However, every paradigm might not be transferable in an experimental setting (eg, falling due to dizziness or collision with another person).

### Conclusions

The re-enactment approach provides a possibility to generate data that is similar to those of real-world fall events. This method could help to better understand real-world falls and further improve the simulation of fall events to increase the available realistic fall data for algorithm development.

## Appendix

Multimedia Appendix 1 Triaxial acceleration signals and timeseries alignment of the vertical component of a further real-world fall event (example 1) and a very similar signal derived during re-enactment experiment.

Multimedia Appendix 2 Triaxial acceleration signals and timeseries alignment of the vertical component of a further real-world fall event (example 2) and a very similar signal derived during re-enactment experiment.

Multimedia Appendix 3 Triaxial acceleration signals and timeseries alignment of the vertical component of a further real-world fall event (example 3) and a very similar signal derived during re-enactment experiment.

## Abbreviations

DTW: dynamic time warping
FARSEEING: Fall Repository for the Design of Smart and Self-Adaptive Environments Prolonging Independent Living
L: lumbar
SG: Samsung Galaxy.
