# Supplementary figures and images for: Re-Enactment as a Method to Reproduce Real-World Fall Events Using Inertial Sensor Data: Development and Usability Study
Source: J Med Internet Res. 2020 Apr 3;22(4):e13961. doi: 10.2196/13961 (PMC7165311; doi:10.2196/13961)

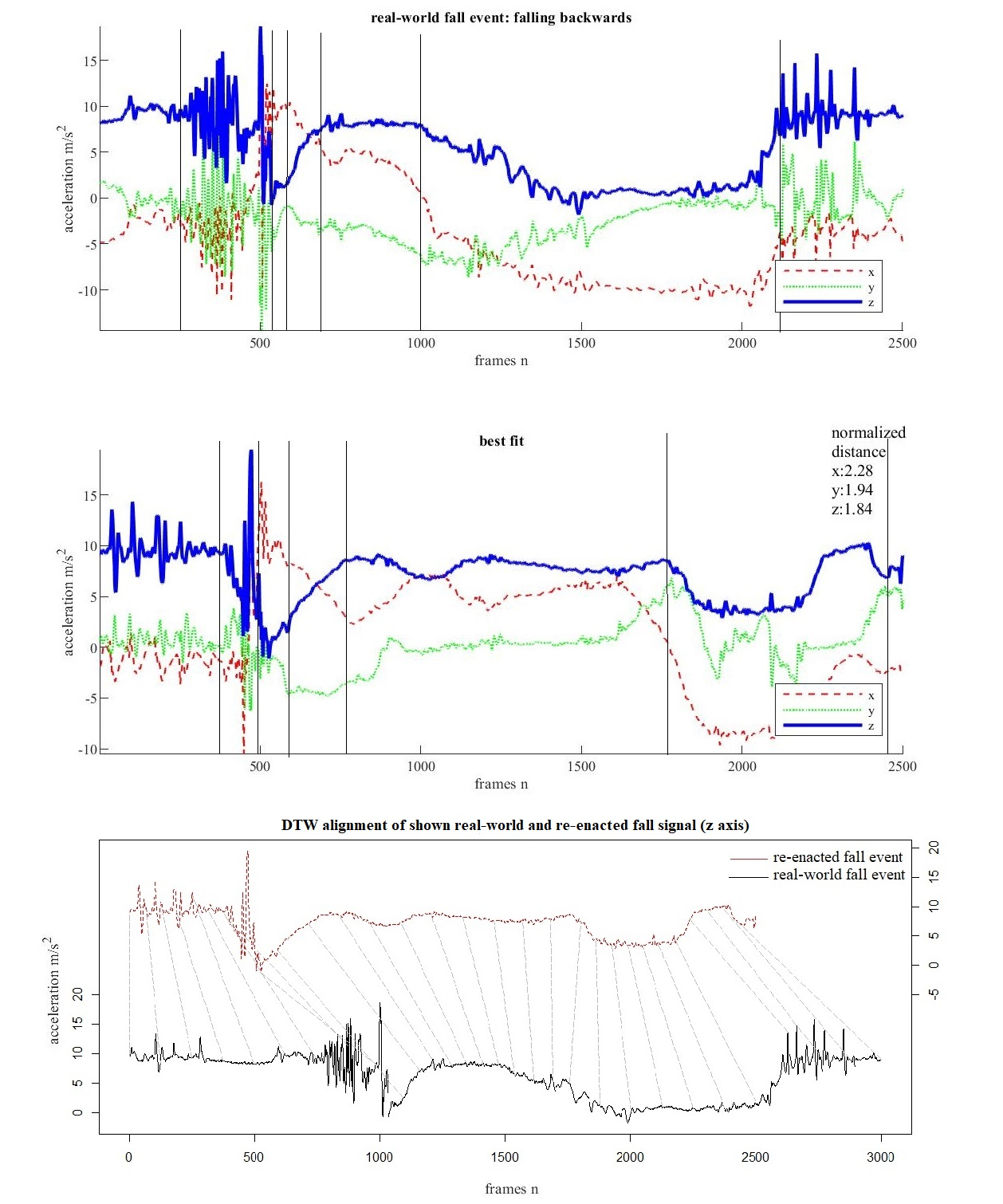

Supplement: Multimedia Appendix 1 [file jmir_v22i4e13961_app1.png]

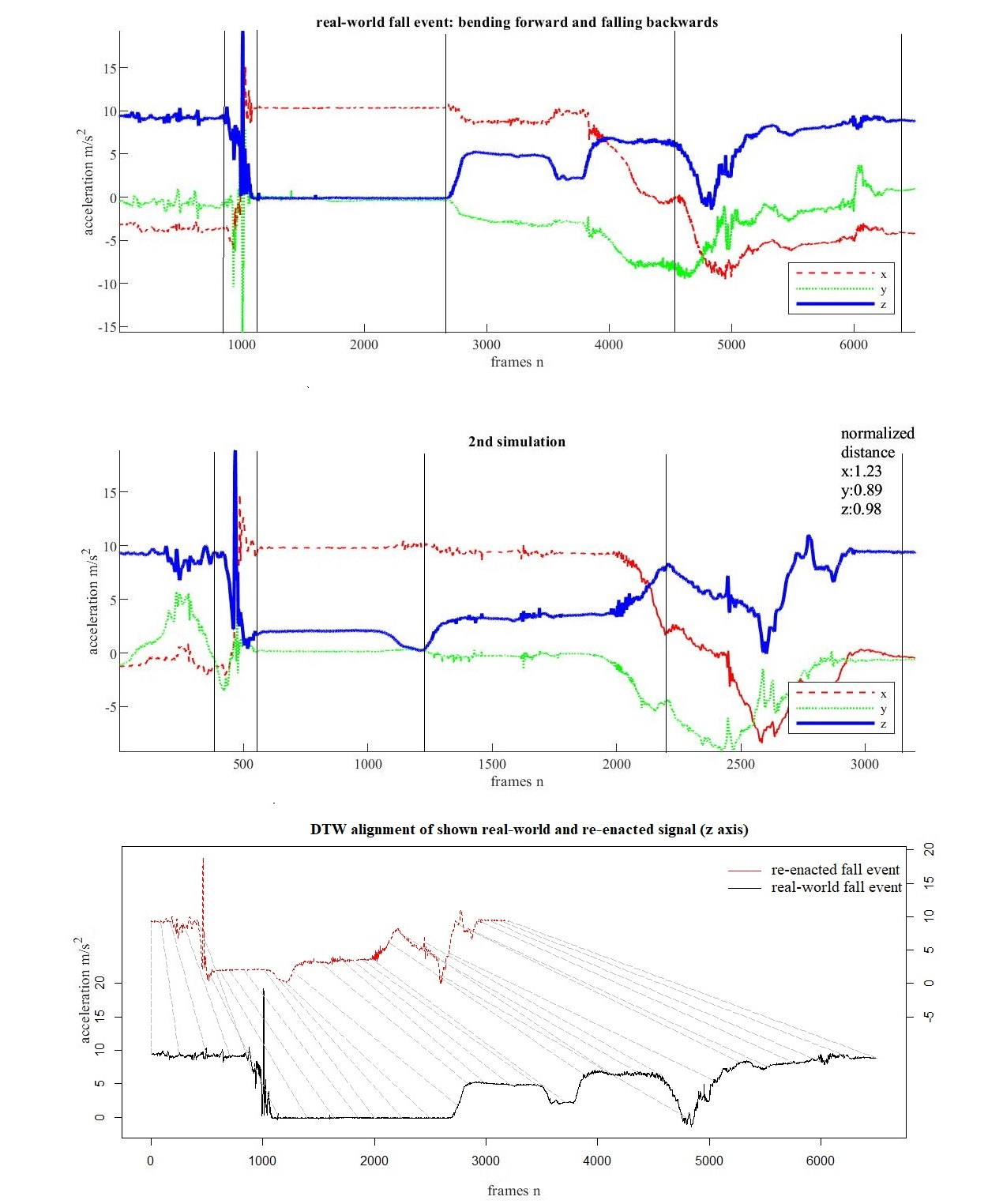

Supplement: Multimedia Appendix 2 [file jmir_v22i4e13961_app2.png]

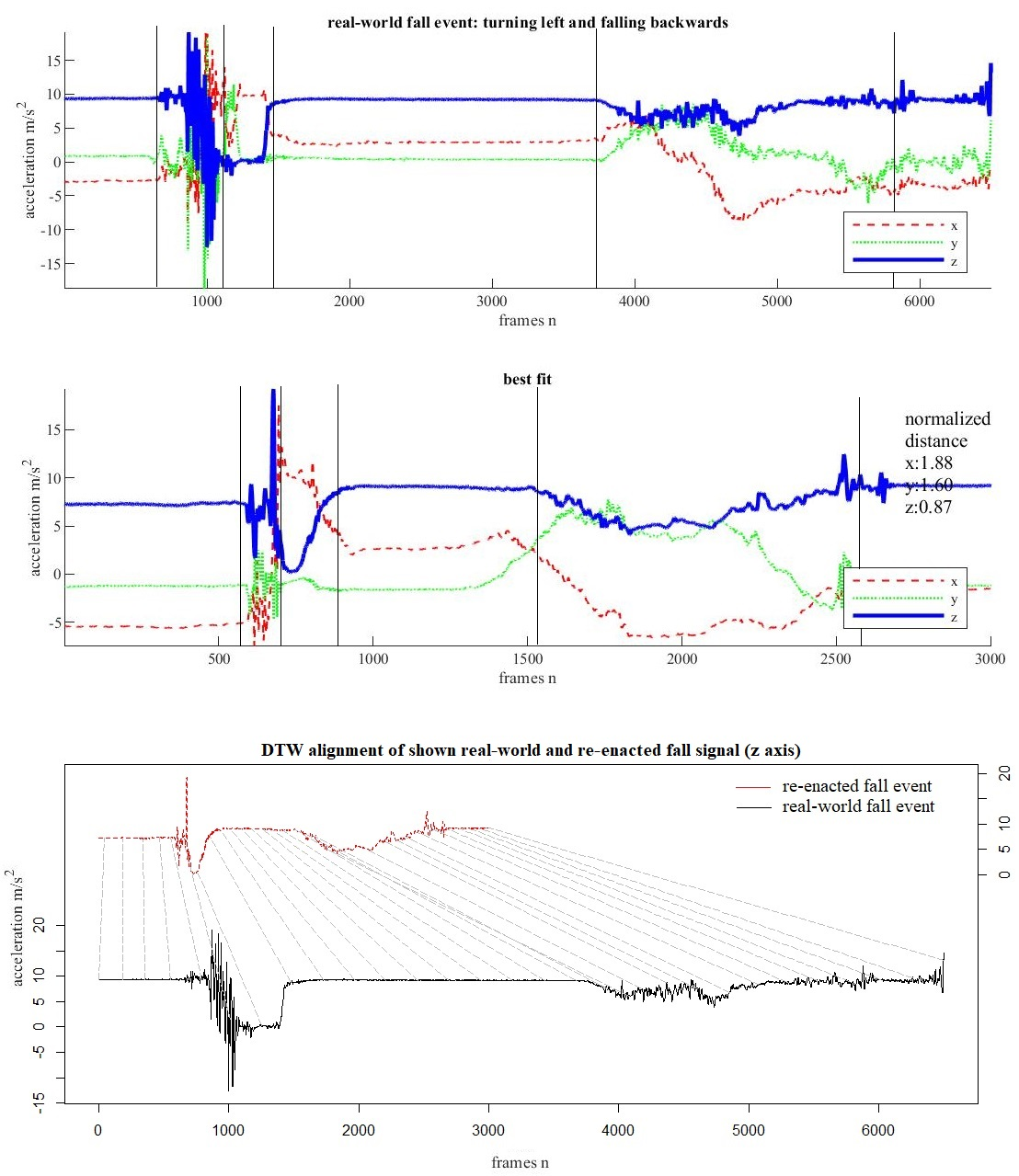

Supplement: Multimedia Appendix 3 [file jmir_v22i4e13961_app3.png]
